# Evaluation of the tuberculin skin test and the interferon-γ release assay for TB screening in French healthcare workers

**DOI:** 10.1186/1745-6673-4-30

**Published:** 2009-11-30

**Authors:** Dominique Tripodi, Benedicte Brunet-Courtois, Virginie Nael, Marie Audrain, Edmond Chailleux, Patrick Germaud, Frederique Naudin, Jean-Yves Muller, Martine Bourrut-Lacouture, Marie-Henriette Durand-Perdriel, Claire Gordeeff, Guyonne Guillaumin, Marietherese Houdebine, Francois Raffi, David Boutoille, Charlotte Biron, Gilles Potel, Claude Roedlich, Christian Geraut, Anja Schablon, Albert Nienhaus

**Affiliations:** 1 Department of Occupational Medicine and Occupational Hazards, University Hospital of Nantes, France; 2 Department of Immunology, University Hospital of Nantes, France; 3 Department of Pneumology, Laënnec Hospital, University Hospital of Nantes, France; 4 Tuberculosis Public Health Clinic, 6 rue Hippolyte Durand Gasselin, Nantes, France; 5 Department of the Infectious and Tropical illnesses, Hospital, CHU Nantes, France; 6 Emergency Department, University Hospital of Nantes, France; 7 Accident Insurance and Prevention in the Health and Welfare Services, Germany

## Abstract

**Introduction:**

Using French cut-offs for the Tuberculin Skin Test (TST), results of the TST were compared with the results of an Interferon-γ Release Assay (IGRA) in Healthcare Workers (HCW) after contact to AFB-positive TB patients.

**Methods:**

Between May 2006 and May 2007, a total of 148 HCWs of the University Hospital in Nantes, France were tested simultaneously with IGRA und TST. A TST was considered to indicate recent latent TB infection (LTBI) if an increase of >10 mm or if TST ≥ 15 mm for those with no previous TST result was observed. For those with a positive TST, chest X-ray was performed and preventive chemotherapy was offered.

**Results:**

All HCWs were BCG-vaccinated. The IGRA was positive in 18.9% and TST ≥ 10 mm was observed in 65.5%. A recent LTBI was believed to be highly probable in 30.4% following TST. Agreement between IGRA and TST was low (kappa 0.041). In 10 (16.7%) out of 60 HCWs who needed chest X-ray following TST the IGRA was positive. In 9 (20%) out of 45 HCWs to whom preventive chemotherapy was offered following TST the IGRA was positive. Of those considered TST-negative following the French guidelines, 20.5% were IGRA-positive. In a two-step strategy - positive TST verified by IGRA - 18 out of 28 (64.3%) IGRA-positive HCWs would not have been detected using French guidelines for TST interpretation.

**Conclusion:**

The introduction of IGRA in contact tracings of BCG-vaccinated HCWs reduces X-rays and preventive chemotherapies. Increasing the cut-off for a positive TST does not seem to be helpful to overcome the effect of BCG vaccination on TST.

## Introduction

The increased risk of healthcare workers (HCWs) for tuberculosis is well established [1,2]. Therefore screening healthcare workers (HCWs) for latent tuberculosis infection (LTBI) and active tuberculosis (TB) is fundamental in infection control programs in hospitals [3]. For about a century, the Tuberculin Skin Test (TST) has been used to detect LTBI. However the TST has its known limitations, including cross-reactivity with BCG and non-tubercular mycobacteria (NTM) infections [4]. Advances in molecular biology have led to the development of new *in-vitro *assays that measure interferon (INF)-γ released by sensitized T-cells after stimulation with *M. tuberculosis *antigens. These tests are more specific than the TST because they use antigens not shared by any of the BCG vaccine strains nor by the more common species of NTM (e.g. *M. avium*) [5]. Besides the higher specificity and at least equal sensitivity as the TST, IGRAs correlate better with surrogate measures of exposure to *M. tuberculosis *[6-8] and have a higher predictive value for LTBI progression to active TB in close contacts in low-incidence settings [9].

So far several systematic investigations of LTBI in HCWs using TST and IGRA have been published [10-18] showing a high proportion of TST-positive/IGRA-negative HCWs which is most likely explained by BCG vaccination. In order to reduce the effect of BCG vaccination on TST, the French guidelines for TST interpretation propose high cut points for the TST - increase >10 mm or ≥ 15 mm if no earlier TST is available [19]. Alternatively to high cut-offs for TST, a number of European Guidelines on the use of IGRAs suggest use of a two-step strategy - performing an IGRA in those initially positive by the TST and excluding LTBI in those IGRA-negative [20-22]. In our study we compared the performance of the TST and IGRA in French HCWs when using a high cut-off for the TST.

## Materials and methods

### Study setting and study subjects

In France the TB incidence in the general population has been declining for several years and was as low as 5.2 cases per 100,000 inhabitants in 2006 [23]. However, increasing differences regarding region and risk groups are seen. Most cases are observed in urban areas and the incidence rate in foreign-born inhabitants was 38.8/100,000).

The population of this cross-sectional study comprises all workers of the University Hospital of Nantes, France, who participated in TB screening from May 2006 through May 2007 because of contact to infectious TB patients or materials. The University Hospital of Nantes is the largest hospital in the Nantes region and serves as a referral center for TB patients throughout the region. Unprotected contact of the HCWs to acid-fast bacillus (AFB)-positive patients occurred in the emergency department and lasted between 1 and 2 hours. Screening was performed 8 to 10 weeks after exposure.

**Figure 1:**
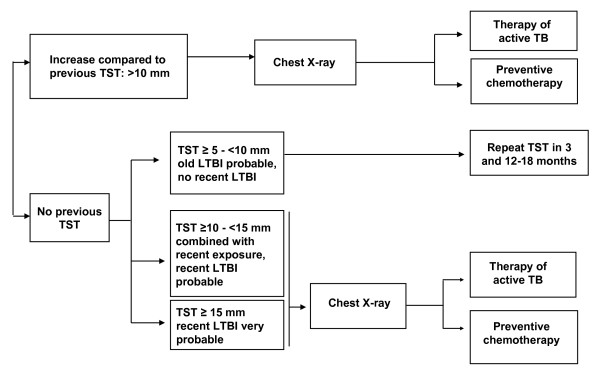
**Decision tree for TST interpretation in contact tracing for contacts of AFB-positive TB patients following **[19]. The French guidelines for TST interpretation in HCWs are presented.

Screening was performed using TST and IGRA simultaneously. Following French guidelines chest X-ray in order to exclude active TB was performed when TST was ≤ 10 mm if no previous TST was available for comparison [19]. If a previous TST was available, X-ray was performed when TST increased by >10 mm (Figure 1). Preventive chemotherapy is proposed if recent LTBI is very probable (TST ≥ 15 mm and no previous TST available or increase >10 mm in TST). For the purpose of this study agreement between IGRA and TST was also analyzed using ≥ 10 mm as cut-off for the TST.

BCG vaccination was assessed through the individual vaccination record or by scars. Following the national vaccination plan, BCG vaccination for newborns is mandatory in France and until 2008 was repeated if TST was <5 mm [23]. Therefore every HCW has been vaccinated at least once. All participating HCWs were French-born.

TST was performed by trained personnel following standard procedures. In brief, 0.1 mL (2 TU) of purified protein derivate (Tubertest from SanofiPasteur) was injected intradermally at the volar side of the forearm and the transverse diameter of the induration was read after 72 to 96 hours [19]. A diameter ≥ 10 mm was considered positive.

Before TST application, the interview was performed and blood for the IGRA was drawn. The interview covered age, gender, BCG vaccination history and employment in healthcare. As IGRA, the QuantiFERON^® ^-TB Gold In-Tube Assay (Cellestis Limited, Carnegie, Australia) was administrated following the manufacturer's protocol. Observers were blinded to the results of the TST and vice versa.

### Statistical analysis

Chi-square tests were used for categorical data. Kappa was calculated for the agreement between IGRA and TST. Adjusted odds ratios (OR) and 95% confidence intervals (CI) were calculated for putative predictive variables using conditional logistic regression. Model building was performed backwards using the chance criteria for variable selection [24].

All persons gave their informed consent prior to their inclusion in the study. No ethics approval of the study was needed because no examinations in addition to those needed for contact tracing were performed.

## Results

The study population comprises 148 HCWs. The characteristics of the study population are described in Table 1. Repeated BCG vaccination had 62.2%. For 83.1% the last vaccination was performed more than 20 years before. No undetermined result of the QFT was observed. A positive QFT was observed in 18.9% and a TST ≥ 10 mm in 65.5% (Table 2). The QFT was positive in 9.8% of those with a TST 0-9 mm and in 21.1% of those with a TST ≥ 20 mm. The association between TST diameter and QFT positivity was weak (p for test for trend: 0.081).

**Table 1:** Description of the study population (n = 148)

Age	N	%
20-29 years	42	28.4

30-39 years	29	19.6

40-49 years	41	27.7

50-60 years	36	24.3

Gender		

Female	109	73.6

Male	39	26.4

BCG vaccination		

One	56	37.8

Two or more	92	62.2

Years since last vaccination		

≤ 20 years	25	16.9

>20-30 years	37	25.0

>30 years	86	58.1

Years in healthcare		

≤ 10 years	78	52.7

>10 years	70	47.3

**Table 2:** TST diameter by IGRA results

	QFT					
	**Negative**		**Positive**		**Total**	

**TST**	**N**	**Row%**	**N**	**Row%**	**N**	**Col%**

0-9 mm	46	90.2	5	9.8	51	34.5

10-14 mm	25	80.2	6	19.4	31	20.9

15-19 mm	19	67.9	9	32.1	28	18.9

20-40 mm	30	78.9	8	21.1	38	25.7

All	120	81.1	28	18.9	148	100.0

P-value for trend: 0.081

TST = Tuberculin Skin Test

QFT = Quantiferon TB Gold in tube

Row% = Row-Percent

Col% = Column-Percent

In 8 persons an increase >10 mm of the TST was observed but only one out of these 8 HCWs (12.5%) had a positive IGRA (Table 3). Most persons (35.1%) had an increase of the TST ≥ 10 mm. 25.0% of the HCWs pertaining to this category were IGRA-positive. No statistically significant association was found between the different TST results and QFT positivity (p = 0.58). Of the 28 HCWs positive in QFT 10 (35.7%) were considered for X-ray or preventive chemotherapy following French interpretation of the TST. Again, following French Guidelines for TST interpretation a recent LTBI was very probable in 45 (30.4%) HCWs (Table 4). Of these, 20% were positive in the QFT which is a proportion similar to the one of those for whom a recent LTBI was not suspected (18.4%). Compared to the French definition for very probable recent LTBI a cut-off of ≥ 10 mm increased the kappa value for agreement between TST and QFT slightly from 0.02 to 0.11. But in both strategies agreement between TST and QFT was weak or non existent.

**Table 3:** QFT results for different TST outcomes

		QFT				
		**Negative**	**Positive**	**Total**		

**TST (p = 0.58)**		**N (row%)**	**N (row%)**	**N (col%)**	**X-ray**	**Chemo**

Increase >10 mm		7 (87.5)	1 (12.5)	8 (5.4)	yes	yes

≥ 15 mm, no earlier TST		29 (78.4)	8 (21.6)	37 (25.0)	yes	yes

≤ 10-<15 mm, no earlier TST		14 (93.3)	1 (6.7)	15 (10.1)	yes	no

≥ 5-<10 mm, no earlier TST		16 (84.2)	3 (15.8)	19 (12.8)	no	no

Increase ≥ 10 mm		39 (75.0)	13 (25.0)	52 (35.1)	no	no

<5 mm, no earlier TST		15 (88.2)	2 (11.8)	17 (11.5)	no	no

Chemo = preventive chemotherapy proposed

TST = Tuberculin Skin Test

QFT = Quantiferon TB Gold in tube

Row% = Row-Percent

Col% = Column-Percent

**Table 4:** Kappa-values and QFT results for TST indicating recent LTBI and TST ≥ 10 mm

	QFT			
	**Negative**	**Positive**	**Total**	

**Recent LTBI very probable**	**N (row%)**	**N (row%)**	**N (col%)**	**Kappa**

No	84 (81.6)	19 (18.4)	103 (69.6)	

Yes	36 (80.0)	9 (20.0)	45 (30.4)	0.02

TST ≥ 10 mm, regardless of earlier TST				

Negative	46 (90.2)	5 (9.8)	51 (34.5)	

Positive	74 (76.3)	23 (23.7)	97 (65.5)	0.11

TST+/QFT- 74 (50% of all), TST-/QFT+ 5 (17.9% of all QFT+)

Using ≥ 10 mm as cut-off for the TST regardless of an earlier TST, TST+/QFT- discordance was observed in 74 (50%) HCWs and TST-/QFT+ discordance in 5 (3.4%) out of 148 exposed HCWs. In those with TST ≥ 10 mm 23.7% were positive in the QFT.

X-ray was performed in 60 HCWs and no active TB was found. Chemoprevention was proposed to 45 HCWs (Table 3). Logistic regression did not reveal any association between positive QFT or positive TST and age, gender, BCG vaccination, or years spent in healthcare. This was also true when instead of the French definitions the cut-off for TST was reduced to ≥ 10 mm regardless of earlier TST results (data not shown).

In a two-step strategy - performing QFT in HCWs with suspected recent LTBI following TST - 10 (1+8+1) instead of 60 (16.7%) would have been proposed for X-ray. On the other side, using this two-step approach with a high cut-off for the TST would allow to detect 10 out of 28 (35.7%) HCWs positive in the QFT only (Table 3). Using a cut-off of ≥ 10 mm for the TST in a two-step strategy would decrease the number of QFTs needed in this population from 100% to 65.5%, again with the drawback that 5 out of 28 (17.9%) HCWs positive in the QFT would be missed (Table 4).

## Discussion

To our knowledge this is the first study that compared the performance of TST and QFT when screening French HCWs. In those in which recent LTBI was suspected following French guidelines [19], the confirmation rate of the QFT was not higher than in those in whom recent LTBI was not probable. Only about one third (35.7%) of those positive in QFT were also considered positive in TST, which is a rate lower than that reported in other studies [10,16,18]. Therefore, our data suggest that the French guidelines for the interpretation of TST in exposed HCWs should be reconsidered. Agreement between TST and QFT was better with ≥ 10 mm as cut-off for TST but still remained weak (kappa = 0.11). Following our data neither increasing the cut-off for TST nor a two-step strategy (IGRA only in TST-positive HCWs) seemed to reduce the influence of BCG vaccination on TST results in a satisfying way.

Positive in QFT were 18.9% of the HCWs with recent contact to an AFB-positive TB case were positive in the QFT. The rate of positive QFT is lower than the one (33%) observed in Portuguese HCWs [25] but lower than the one (10%) observed in German HCWs [16,18].

As reported in another study [26] we observed an association between the diameter of the TST and the probability of a positive QFT even though the test for trend was of borderline statistical significance. This might be due to the small sample size (n = 148) of our study. The number of TST-/QFT+ HCWs in our population is much higher than in two meta-analyses [8,26]. But surprisingly, even though the criteria for a positive TST following French guidelines are high compared to other countries, the proportion of TST+/QFT- HCWs was high, too. The risk of progression to active TB in TST+/QFT- HCWs is unknown. A number of publications suggest it is low [9,27-29]. Because of the high sensitivity of the IGRA and the low specificity of the TST, none of the several national guidelines recommend X-ray or chemoprevention in HCWs with a positive TST and a negative IGRA [20-22]. Therefore it seems reasonable to assume that most TST+/QFT- results do not indicate infection with *Mycobacteria tuberculosis*.

Introducing IGRA for TB screening in France would reduce the number of X-rays and the preventive chemotherapies by a high proportion (from 65.5% for TST ≥ 10 mm or from 40.5% for TST interpretation following French guidelines to 18.9%). We did not conduct a cost-effectiveness analysis. But nevertheless our analysis corroborates the findings of other cost-benefit analyses. A German cost-effectiveness analysis based on German data on TST and QFT positivity and costs of treatment showed that using the QFT assay, but especially combining the QFT assay following the TST screening of close contacts at a cut-off induration size of 5 mm before LTBI treatment, is highly cost-effective in reducing the disease burden of TB [30]. Similar results were observed in an analysis of a hypothetical cohort based on data from Switzerland [31] and Canada [32]. There is still an ongoing debate as to whether the introduction of IGRA in TB screening reduces the costs for BCG-vaccinated contacts only [33] or for both vaccinated and non-vaccinated contacts [34]. It is still unknown for how long the QFT remains positive after elimination of *Mycobacterium tuberculosis *from the body either spontaneously or after chemoprevention [35,36].

So far the risk of progression towards active tuberculosis for those with TST-/QFT+ combinations is unknown. The well-established high specificity of the QFT and the known limitations of TST sensitivity [8] suggest that this combination indicates infections with *Mycobacterium tuberculosis*. Following our data a two-step strategy using ≥ 10 mm as cut-off for a positive TST helps to reduce unwarranted X-rays but risks to miss a high proportion (17.9%) of the HCWs positive in QFT and therefore likely infected with *Mycobacterium tuberculosis*. Further reducing the cut-off for a positive TST to >5 mm would not be useful in our population because already 65% had a TST ≥ 10 mm.

## Conclusion

Our data show that the increase criteria (increase >10 mm) for TST interpretation lead to a low sensitivity of the TST without reducing the specificity problems of the TST in a meaningful way. Therefore the use of the increase criteria should be reconsidered. Furthermore it could be shown that a two-step strategy - IGRA if TST ≥ 10 mm - might also lead to a low sensitivity of a TB screening. Therefore our data suggest that IGRA should replace TST when screening HCWs for tuberculosis.

## Competing interests

The authors declare that they have no competing interests.

## Authors' contributions

DT designed the study and was involved in data collection and writing of the paper.

BBC was involved in data collection and gave critical comments for manuscript writing.

VN was involved in data collection and gave critical comments for manuscript writing.

MA was involved in data collection and gave critical comments for manuscript writing.

EC was involved in data collection and gave critical comments for manuscript writing.

PG was involved in data collection and gave critical comments for manuscript writing.

FN was involved in data collection and gave critical comments for manuscript writing.

JYM was involved in data collection and gave critical comments for manuscript writing.

MBL was involved in data collection and gave critical comments for manuscript writing.

MHDP was involved in data collection and gave critical comments for manuscript writing.

CG was involved in data collection and gave critical comments for manuscript writing.

GG was involved in data collection and gave critical comments for manuscript writing.

MTH was involved in data collection and gave critical comments for manuscript writing.

FR was involved in data collection and gave critical comments for manuscript writing.

DB was involved in data collection and gave critical comments for manuscript writing.

CB was involved in data collection and gave critical comments for manuscript writing.

GP was involved in data collection and gave critical comments for manuscript writing.

CR was involved in data collection and gave critical comments for manuscript writing.

CG was involved in data collection and gave critical comments for manuscript writing.

AN analysed the data and drafted the manuscript.

All authors read and approved the final manuscript.
